# Recognition of Lipoproteins by Toll-like Receptor 2 and DNA by the AIM2 Inflammasome Is Responsible for Production of Interleukin-1β by Virulent Suilysin-Negative *Streptococcus suis* Serotype 2

**DOI:** 10.3390/pathogens9020147

**Published:** 2020-02-21

**Authors:** Agustina Lavagna, Jean-Philippe Auger, Stephen E. Giradin, Nicolas Gisch, Mariela Segura, Marcelo Gottschalk

**Affiliations:** 1 Research Group on Infectious Diseases in Production Animals & Swine and Poultry Infectious Diseases Research Centre, Faculty of Veterinary Medicine, University of Montreal, St-Hyacinthe, QC J2S 2M2, Canada; agustina.lavagna@mcgill.ca (A.L.); jean-philippe.auger.1@umontreal.ca (J.-P.A.); mariela.segura@umontreal.ca (M.S.); 2 Department of Laboratory Medicine and Pathobiology, University of Toronto, Toronto, ON M58 1A8, Canada; stephen.girardin@utoronto.ca; 3 Division of Bioanalytical Chemistry, Priority Area Infections, Research Center Borstel, Leibniz Lung Center, 23845 Borstel, Germany; ngisch@fz-borstel.de

**Keywords:** *Streptococcus suis*, interleukin-1, dendritic cell, suilysin-negative, Toll-like receptor 2, lipoprotein, AIM2 inflammasome, inflammation, septic shock

## Abstract

*Streptococcus suis* serotype 2 is an important porcine bacterial pathogen and zoonotic agent causing sudden death, septic shock and meningitis. These pathologies are the consequence of an exacerbated inflammatory response composed of various mediators including interleukin (IL)-1β. Elevated levels of the toxin suilysin (SLY) were demonstrated to play a key role in *S. suis*-induced IL-1β production. However, 95% of serotype 2 strains isolated from diseased pigs in North America, many of which are virulent, do not produce SLY. In this study, we demonstrated that SLY-negative *S. suis* induces elevated levels of IL-1β in systemic organs, with dendritic cells contributing to this production. SLY-negative *S. suis*-induced IL-1β production requires MyD88 and TLR2 following recognition of lipoproteins. However, the higher internalization rate of the SLY-negative strain results in intracellularly located DNA being recognized by the AIM2 inflammasome, which promotes IL-1β production. Finally, the role of IL-1 in host survival during the *S. suis* systemic infection is beneficial and conserved, regardless of SLY production, via modulation of the inflammation required to control bacterial burden. In conclusion, this study demonstrates that SLY is not required for *S. suis*-induced IL-1β production.

## 1. Introduction

*Streptococcus suis* is an important porcine bacterial pathogen associated with meningitis, sepsis, arthritis, and endocarditis, among other pathologies [1]. Additionally, *S. suis* is a zoonotic agent, responsible for hundreds of human cases annually, particularly in Southeast Asia [2,3]. Of the described serotypes based on the capsular polysaccharide (CPS) antigens, serotype 2 is the most frequently isolated from diseased pigs and humans worldwide [4]. However, serotype 2 strains are highly heterogeneous and belong to numerous sequence types (STs), as determined using multilocus sequence typing, with the highly virulent ST1 predominating in Eurasia, the epidemic virulent ST7 in China and the virulent ST25 in North America [5]. Low virulence ST28 strains are also frequently isolated in the United States [6]. Furthermore, isolates with variable virulence belonging to the latter two STs have also been reported in Asia [4,7,8,9]. While generally considered less virulent than ST1 strains (which only represent 5% of serotype 2 strains in North America), ST25 strains are frequently isolated from diseased pigs in Canada [10].

The *S. suis* pathogenesis and subsequent host response have been partially characterized, with a variety of virulence factors described [11]. The CPS confers anti-phagocytic properties important for systemic persistence and dissemination, while certain strains produce a hemolysin, termed suilysin (SLY), involved in modulating the interactions with host cells and their inflammatory response [11]. Finally, bacterial components such as lipoproteins (LPs) and lipoteichoic acid (LTA) have also been suggested to be involved in the *S. suis* pathogenesis [12,13].

Initial recognition of *S. suis* by innate immune cells involves specialized membrane-associated or cytoplasmic receptors (pattern recognition receptors (PRRs)), which include Toll-like receptor (TLR) 2, TLR4, TLR7, and TLR9, as well as the adaptor protein myeloid differentiation primary response 88 (MyD88) [14,15]. Their activation leads to the synthesis of diverse pro-inflammatory mediators via recruitment of the nuclear factor-kappa B (NF-ĸB) and mitogen-activated protein kinases (MAPKs) [16,17]. Of the different innate cells involved, dendritic cells (DCs) are required for the control and elimination of *S. suis* via its phagocytosis and participate in the induced inflammatory response [18,19]. Indeed, DCs are important sources of various pro-inflammatory mediators including interleukin (IL)-1 following *S. suis* infection [13,14,19].

IL-1, one of the most potent and earliest pro-inflammatory mediators produced, is involved in the recruitment of inflammatory cells, their activation and induction of other inflammatory factors [20,21,22,23]. Its two forms, IL-1α and IL-1β bind the shared IL-1 receptor (IL-1R), which is ubiquitously expressed, resulting in the synthesis of inflammatory mediators, adhesion molecules and acute phase proteins [24]. IL-1β is synthesized as a precursor peptide (pro-IL-1β) requiring a two-step processing mechanism for production [22,25]. Firstly, activation of PRRs leads to the transcription and translation of pro-IL-1β, which is then cleaved to become active, mainly via caspase-1-dependent mechanisms [26]. Similarly to pro-IL-1β, caspase-1 itself requires proteolytic processing, which is mediated by inflammasomes, with the nucleotide-binding oligomerization domain (NOD)-like receptor (NLR) family pyrin domain-containing 3 (NLRP3), the NLRP1, the NLR family CARD domain-containing protein 4 (NLRC4), and the absent in melanoma 2 (AIM2) being the best characterized [27,28]. 

Although IL-1 signaling is essential for immunity by participating in the initiation of the inflammatory response, an uncontrolled production of IL-1 can lead to tissue damage and disease. Indeed, IL-1 plays a protective role during both pneumococcal and Group B *Streptococcus* infections, during which a lack of IL-1 signaling dampens the inflammatory response, resulting in increased bacterial burden [23,29,30,31]. Conversely, a lack of IL-1β production is lethal in a mouse model of Group A *Streptococcus* infection [32,33]. Moreover, IL-1 signaling was recently demonstrated to play a beneficial role during the systemic infection caused by a highly virulent *S. suis* serotype 2 ST1 strain via initiation of the inflammatory cascade and promotion of bacterial clearance [13]. However, this effect was not observed following infection with the epidemic ST7 strain responsible for the 2005 human outbreak due to the exacerbated inflammation being too elevated for counterbalancing by IL-1 [13,34]. The mechanism presently described for *S. suis*-induced IL-1β production involves SLY (both ST1 and ST7 strains are SLY-positive), which promotes its processing [13,34]. However, a large proportion of virulent *S. suis* serotype 2 strains recovered from diseased animals do not produce SLY, including the virulent ST25 strains present in Canada and Thailand [10,35], and their capacity to produce IL-1β, including the mechanisms involved, have been little studied. Consequently, IL-1 production induced by a virulent SLY-negative *S. suis* serotype 2 ST25 strain was further characterized in vitro and the role of IL-1 signaling induced by this strain determined during the systemic infection in vivo.

## 2. Results

### 2.1. Suilysin-Negative S. suis Induces Elevated Levels of IL-1β in Spleen and Liver But Not in Plasma

**Figure 1:**
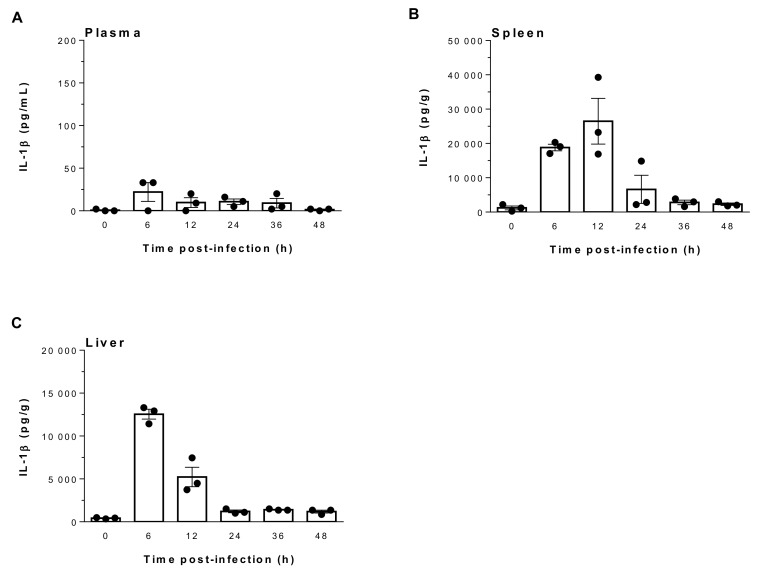
Suilysin (SLY)-negative *S. suis* strain 89-1591 induces elevated levels of IL-1β production in spleen and liver but not in plasma. C57BL/6 mice were intraperitoneally inoculated with 1 × 10^7^ CFU, plasma (**A**), spleen (**B**) and liver (**C**) were collected at indicated times post-infection and IL-1β levels were quantified by ELISA. Values for mock-infected controls remained constant from 6 h to 48 h. As such, 0 h represents results for mock-infected mice. Data are expressed as mean ± SEM (*n* = 3).

*S. suis* infection and dissemination can lead to the production of inflammatory mediators, including IL-1β [13,36], which is a key cytokine initiating the inflammatory cascade. Consequently, its production in plasma, spleen and liver was evaluated at different incubation times following infection with the virulent SLY-negative ST25 strain 89-1591. Since IL-1β levels in plasma, spleen and liver of mock-infected mice were barely detectable and remained constant from 6 h to 48 h, 0 h represents results for non-infected mice (Figure 1). Infection with strain 89-1591 failed to induce a robust IL-1β response in plasma, regardless of time, with values lower than 25 pg/mL (Figure 1A). However, IL-1β production in liver and spleen was elevated, with the highest levels at 6 h and 12 h post-infection (p.i.) (Figure 1B,C). These results demonstrate that though unable to produce SLY, *S. suis* strain 89-1591 (ST25) induces an elevated IL-1β response in systemic organs but not in plasma.

### 2.2. Suilysin-Negative S. suis Induces Elevated IL-1β Production From Bone-Marrow Dendritic Cells in a Time-Dependent Manner

**Figure 2:**
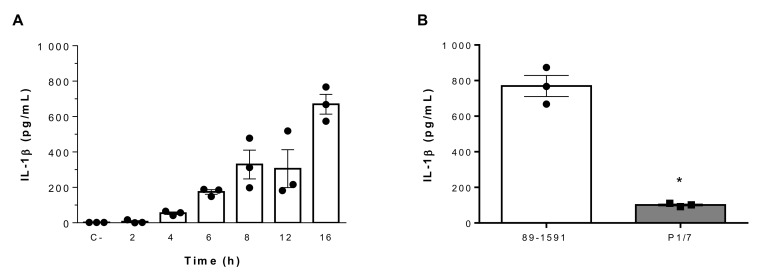
Kinetics of IL-1β production from bone marrow-derived dendritic cells (bmDCs) infected with SLY-negative *S. suis* strain 89-1591 and comparison to SLY-positive strain P1/7. (**A**) Strain 89-1591-induced IL-1β production kinetic from bmDCs as measured by ELISA. Non-stimulated cells served as negative control (C-). (**B**) bmDCs were infected with 1 × 10^6^ CFU of strain 89-1591 or P1/7 for 16 h and IL-1β release was measured by ELISA. Data are expressed as mean ± SEM (*n* = 3). * (*p* < 0.05) indicates a significant difference between strains.

The elevated IL-1β response induced by *S. suis* strain 89-1591 in spleen and liver suggests a role of resident innate immune cells, of which DCs are involved during *S. suis* infection and are an important source of IL-1β [13,14,19]. Consequently, the capacity of these cells to produce IL-1β following infection with the SLY-negative strain 89-1591 was evaluated using bone marrow-derived dendritic cells (bmDCs), which are a well-characterized model of conventional DCs [13,14,18,19,37]. Strain 89-1591 induced IL-1β production in a time-dependent manner (Figure 2A). Though incapable of producing SLY, strain 89-1591 nonetheless induced significantly higher levels of IL-1β from bmDCs than those induced by the highly virulent SLY-positive ST1 strain P1/7 (*p* < 0.05) (Figure 2B). 

### 2.3. Role of Toll-Like Receptors and Associated Signaling Pathways in Suilysin-Negative S. suis-Induced IL-1β Production

**Figure 3:**
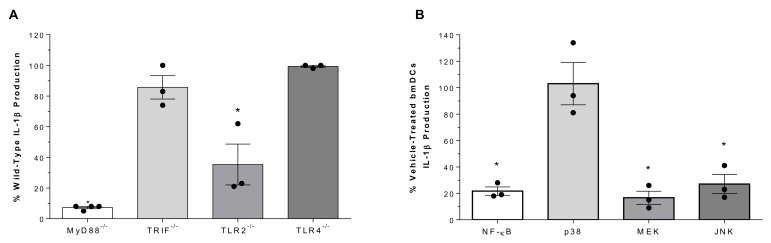
Role of TLRs and associated signaling in SLY-negative *S. suis* strain 89-1591-induced IL-1β production by bmDCs. (**A**) Percentage of IL-1β production induced 16 h following infection of bmDCs deficient for MyD88, TRIF, TLR2 or TLR4, with regards to wild-type counterparts (normalized to 100%). (**B**) Percentage of IL-1β production from bmDCs following pretreatment with inhibitors of NF-kB, p38, MEK or JNK and infection with 1 × 10^6^ CFU of strain 89-1591 with regards to vehicle-treated bmDCs (DMSO; normalized to 100%). Data are expressed as mean ± SEM (*n* = 3). * (*p* < 0.05) indicates a significant difference with wild-type or vehicle-treated bmDCs.

Different cellular pathways are involved in bacterial recognition, with the TLR pathway crucial for *S. suis* [14,19]. Production of IL-1β induced by the SLY-negative strain 89-1591 was almost completely abrogated in the absence of the adaptor protein MyD88 (*p* < 0.01) but was unaffected by the absence of TIR-domain-containing adapter-inducing IFN-β (TRIF) (Figure 3A). Since *S. suis* is mostly an extracellular pathogen, its recognition by surface-associated receptors is crucial. While IL-1β production was significantly reduced in TLR2^-/-^ bmDCs (*p* < 0.01), no difference was observed with TLR4^-/-^ DCs (Figure 3A).

The NF-κB and MAPK pathways are implicated in the transcriptional control of IL-1β [38,39]. Consequently, bmDCs were pre-treated with different inhibitors (NF-κB inhibitor [i] JSH-23, p38i SB203580, MEK1/2i U0126 or JNKi SP600125) or the vehicle (dimethylsulfoxide; DMSO). While inhibition of NF-κB, MEK1/2 and JNK significantly and equally reduced IL-1β production (*p* < 0.01), p38 inhibition had no effect (Figure 3B). These data indicate that SLY-negative *S. suis* strain 89-1591-induced IL-1β is MyD88-dependent, requiring TLR2, and signals via NF-κB, MEK1/2 and JNK.

### 2.4. Suilysin-Negative S. suis Lipoproteins and Nucleic Acids Are Potent Inducers of IL-1β Production

**Figure 4:**
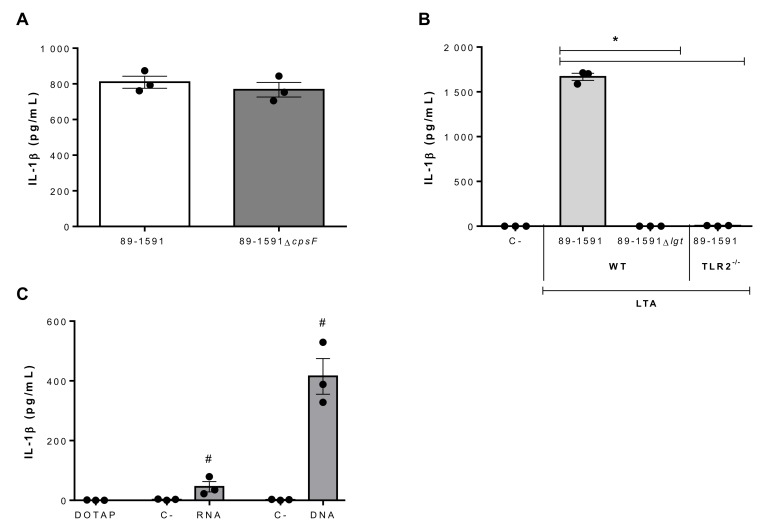
Role of SLY-negative *S. suis* strain 89-1591 components in bmDC-produced IL-1β. (**A**) IL-1β production by bmDCs following 16 h of infection with 1 × 10^6^ CFU of strain 89-1591 or its capsular polysaccharide-deficient mutant (89-1591∆*cpsF*). (**B**) IL-1β production 24 h following activation of wild-type (WT) or TLR2^-/-^ bmDCs with 30 μg/mL of lipoteichoic acid (LTA) extracts from strain 89-1591 or its *lgt*-deficient mutant (89-1591Δ*lgt*). Non-stimulated cells served as negative control (C-). (**C**) IL-1β production by bmDCs following phagosomal delivery of 1 μg of *S. suis* RNA or DNA. Cells stimulated with elution buffer served as negative control (C-). Data are expressed as mean ± SEM (*n* = 3). * (*p* < 0.05) indicates a significant difference with 89-1591-derived LTA and # (*p* < 0.05) with the negative control.

Though a multitude of *S. suis* components have been described to induce pro-inflammatory mediator production from innate immune cells, the presence of CPS is usually associated with partial masking of these components [11,12]. However, this was previously reported not to be the case for strain 89-1591 [40]. In accordance, no difference was observed in IL-1β production between strain 89-1591 and its non-encapsulated isogenic mutant, 89-1591Δ*cpsF* (Figure 4A).

Given the implication of TLR2 in strain 89-1591-induced IL-1β production, potential activators were investigated. LTA and LPs have been suggested to activate TLR2 in Gram-positive bacteria [41,42,43]. Consequently, LTA was extracted and bmDCs stimulated, inducing high levels of IL-1β production (Figure 4B). As previously described, however, current methods are unable to eliminate co-purified LPs from LTA preparations [42]. As such, LTA was also extracted from *lgt*-deficient mutants (Δ*lgt*), in which absence of the lipoprotein diacylglyceryl transferase, a key enzyme in LP synthesis, renders LPs biological inactive and unrecognizable by TLR2 [44,45]. In accordance, not only did LTA preparations from *lgt*-deficient mutants induce significantly less IL-1β than those from the wild-type strain (*p* < 0.01), but levels were undetectable (Figure 4B). In addition, IL-1β production was completely abolished in TLR2^-/-^ bmDCs following activation with LTA preparations from the wild-type strain (*p* < 0.01) (Figure 4B). Taken together, these results indicate that strain 89-1591 LPs are important inducers and mainly responsible for IL-1β production by bmDCs via recognition by TLR2.

Dependence of *S. suis*-induced IL-1β production on MyD88, but only partially on TLR2 and not at all on TLR4, suggested a co-participation of endosomal TLRs. In fact, it was previously demonstrated that when internalized*, S. suis* nucleic acids can induce bmDC activation [19]. As such, DNA and RNA were extracted from strain 89-1591 and complexed or not with DOTAP liposomal transfection reagent, which allows phagosomal delivery. DNA and RNA induced IL-1β production from bmDCs only when complexed with DOTAP (*p* < 0.05), though DNA was significantly more stimulating than RNA (*p* < 0.01) (Figure 4C). Consequently, this recognition of RNA and DNA might suggest the involvement of TLR7 and TLR9, respectively. 

### 2.5. Suilysin-Negative S. suis-Induced IL-1β Production Depends on Caspase-1 and the NLRP3 and AIM2 Inflammasomes

**Figure 5:**
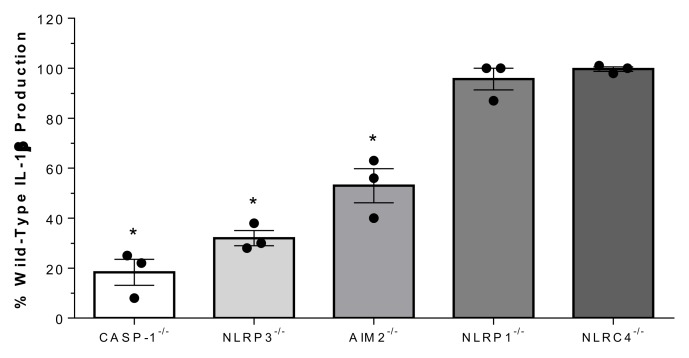
SLY-negative *S. suis* strain 89-1591-induced bmDC IL-1β production is caspase-1, NLRP3 and AIM2 dependent. Percentage of IL-1β secretion by caspase-1- (CASP-1), NLRP3-, AIM2-, NLRP1- or NLRC4-deficient bmDCs after 16 h of infection with 1 × 10^6^ CFU of strain 89-1591 by ELISA, in comparison to wild-type counterparts (normalized to 100%). Data represent the mean ± SEM (*n* = 3); * (*p* < 0.05) indicates a significant difference obtained with wild-type bmDCs.

Processing of IL-1β requires cleavage by proteolytic enzymes, the most important of which is caspase-1 [24,25]. To investigate whether strain 89-1591-induced IL-1β requires this enzyme, caspase-1-deficient bmDCs were used. As shown in Figure 5, IL-1β production was reduced by more than 75% in caspase-1^-/-^ bmDCs (*p* < 0.01). To determine the mechanisms by which strain 89-1591 might activate caspase-1, the role of the NLRP1, NLRP3, AIM2, and NLRC4 inflammasomes, which are the best characterized [27], was evaluated. While NLRP3- and AIM2-deficiency resulted in a significant decrease of strain 89-1591-induced IL-1β production (*p* < 0.05), NLRP1 and NLRC4 were not involved (Figure 5). 

### 2.6. S. suis Strain 89-1591-Induced IL-1β Production Requires Internalization and Intracellular DNA Sensing by the AIM2 Inflammasome

**Figure 6:**
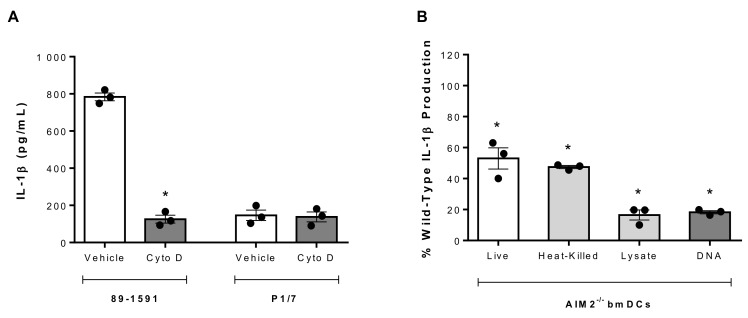
SLY-negative *S. suis* strain 89-1591-induced bmDC IL-1β production involves internalization and recognition of bacterial DNA by AIM2 inflammasome. (**A**) IL-1β production from bmDCs following pretreatment with vehicle (DMSO) or cytochalasin D (Cyto D) and infection with 1 × 10^6^ CFU of strain 89-1591 or strain P1/7 for 16 h. (**B**) Percentage of IL-1β production in AIM2^-/-^ bmDCs stimulated with live (1 × 10^6^ CFU) or whole heat-killed strain 89-1591 (1 × 10^9^ CFU), 5 µg of strain 89-1591 lysate or 1 µg of strain 89-1591 DNA, in comparison to wild-type counterparts (normalized to 100%), as measured by ELISA after 16 h. Data represent the mean ± SEM (*n* = 3). * (*p* < 0.05) indicates a significant difference with vehicle-treated or wild-type bmDCs.

The greater IL-1β production by the SLY-negative strain 89-1591 from bmDCs than the SLY-positive strain P1/7 suggested differential processing mechanisms. A notable difference between the interactions of these strains with bmDCs is the significantly greater internalization of strain 89-1591, regardless of its high encapsulation [19]. Consequently, bmDC internalization was blocked by pretreatment with cytochalasin D, which inhibits actin polymerization, or its vehicle (DMSO), as previously described [19]. Inhibiting internalization significantly reduced 89-1591-induced IL-1β production by bmDCs (*p* < 0.05) (Figure 6A). By contrast, no difference in P1/7-induced IL-1β production was observed following inhibition of internalization (Figure 6A). Moreover, the involvement of the NLRP3 and AIM2 inflammasomes described above is greater for strain 89-1591 than that previously published for strain P1/7 [13], which was confirmed herein (data not shown). While the bacterial components responsible for NLRP3 activation other than secreted toxins remain little studied [46], double-stranded DNA has been described to be sensed by the AIM2 inflammasome [47,48]. Consequently, AIM2^-/-^ bmDCs were used to better understand the underlying mechanisms. Activation of AIM2^-/-^ bmDCs with whole heat-killed 89-1591 resulted in a similar IL-1β production as with live bacteria (Figure 6B), suggesting that the motif recognized by the AIM2 inflammasome is not secreted. Subsequently, strain 89-1591 was lysed using sonication and lysates were complexed or not with DOTAP. In the absence of the AIM2 inflammasome, production of IL-1β using lysates was significantly lower than with live or heat-killed bacteria (*p* < 0.05) (Figure 6B), but only when complexed with DOTAP. Finally, activation of AIM2^-/-^ bmDCs with strain 89-1591 DOTAP-complexed DNA resulted in a near complete abolishment of IL-1β production (*p* < 0.05), similar to the bacterial lysate (Figure 6B), while no difference was observed using RNA (data not shown). Taken together, these results demonstrate that intracellularly located *S. suis* DNA is recognized by the AIM2 inflammasome and that the higher internalization rate of strain 89-1591 by bmDCs results in a more efficient processing of the induced IL-1β. 

### 2.7. IL-1 Signaling Plays a Beneficial Role in Host Survival During the Systemic Infection Induced by Suilysin-Negative S. suis

**Figure 7:**
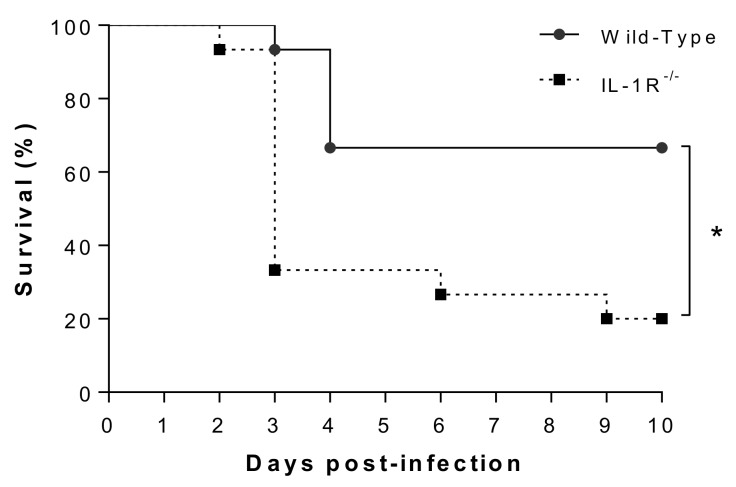
Survival of wild-type and IL-1R^-/-^ mice after intraperitoneal infection with SLY-negative *S. suis* strain 89-1591. Six-week-old mice were intraperitoneally inoculated with 1 × 10^7^ CFU of strain 89-1591 and survival was monitored for 10 days post-infection. Data represent survival curves (*n* = 15). * (*p* < 0.05) indicates a significant difference between survival of wild-type and IL-1R^-/-^ mice.

Due the importance of IL-1 in the balance of systemic inflammation [13,34], host survival following SLY-negative *S. suis* strain 89-1591 infection was evaluated in wild-type and IL-1R^-/-^ mice. Survival of IL-1R^-/-^ mice was significantly reduced in comparison to wild-type counterparts (*p* < 0.01) (Figure 7), suggesting a beneficial role of IL-1 signaling during SLY-negative strain 89-1591 infection.

**Figure 8:**
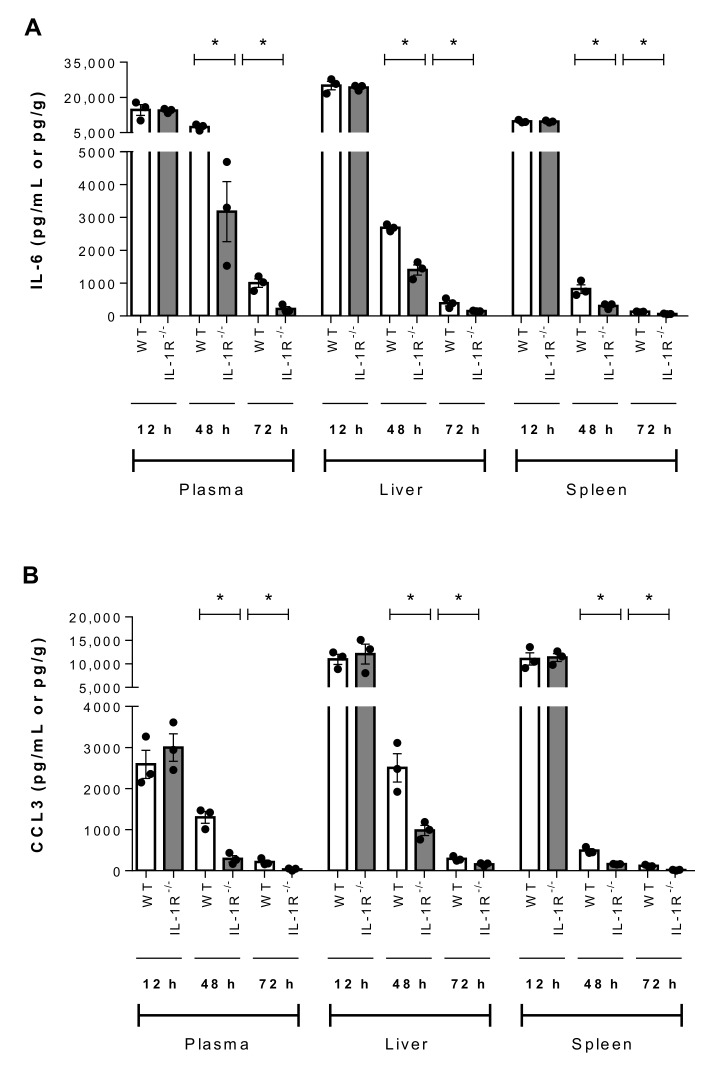
IL-1 is required for SLY-negative *S. suis* strain 89-1591-induced IL-6 and CCL3 production in blood, liver and spleen. Plasma, liver and spleen levels of IL-6 (**A**) and CCL3 (**B**) in wild-type (WT) and IL-1R^-/-^ mice 12 h, 48 h and 72 h following infection with 1 × 10^7^ CFU. Data represent the mean ± SEM (*n* = 3). * (*p* < 0.05) indicates a significant difference between WT and IL-1R^-/-^ mice.

**Figure 9:**
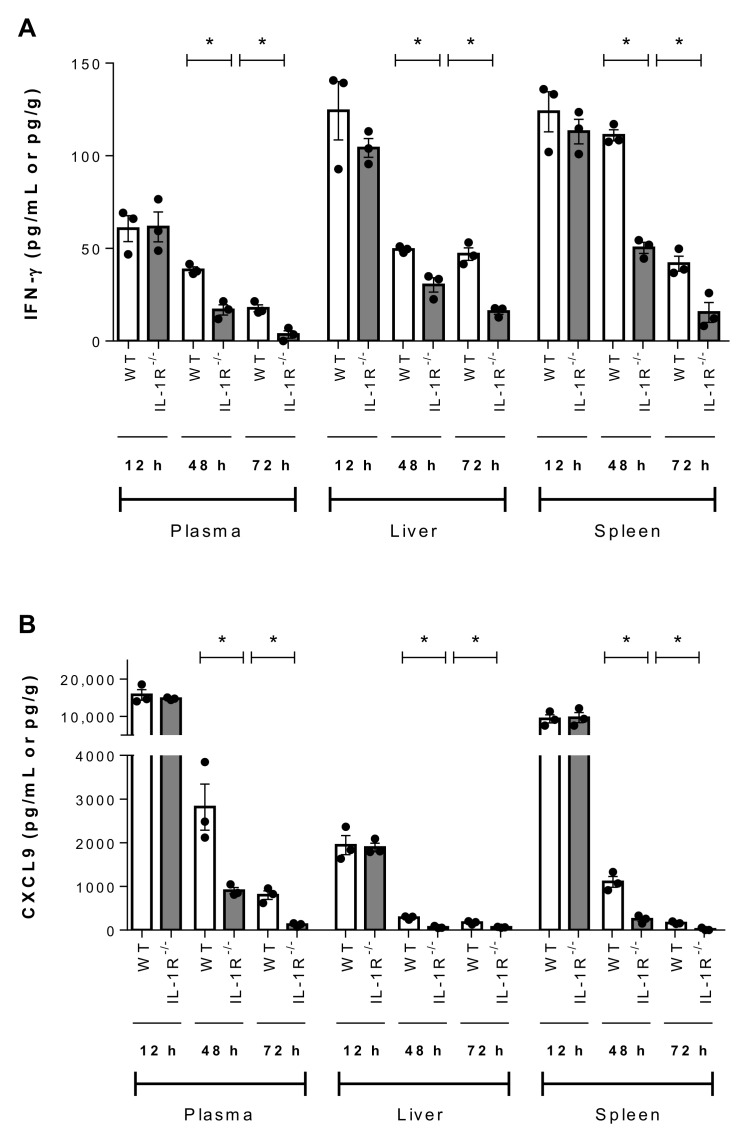
IL-1 is required for SLY-negative *S. suis* strain 89-1591-induced IFN-γ and CXCL9 production in blood, liver and spleen. Plasma, liver and spleen levels of IFN-γ (**A**) and CXCL9 (**B**) in wild-type (WT) and IL-1R^-/-^ mice 12 h, 48 h and 72 h following infection with 1 × 10^7^ CFU. Data represent the mean ± SEM (*n* = 3). * (*p* < 0.05) indicates a significant difference between WT and IL-1R^-/-^ mice.

Since IL-1 signaling is involved in initiation of the inflammatory cascade, the production of other pro-inflammatory mediators (IL-6, IFN-γ, C-C motif chemokine ligand (CCL) 3, and C-X-C motif chemokine ligand (CXCL) 9) in plasma, liver and spleen was evaluated 12 h, 48 h, and 72 h p.i. While no differences were observed at 12 h, significantly lower levels of IL-6, IFN-γ, CCL3, and CXCL9 were observed 48 h and 72 h p.i. in the plasma, liver and spleen of IL-1R^-/-^ mice than in wild-type counterparts (*p* < 0.05) (Figure 8 and Figure 9).

**Figure 10:**
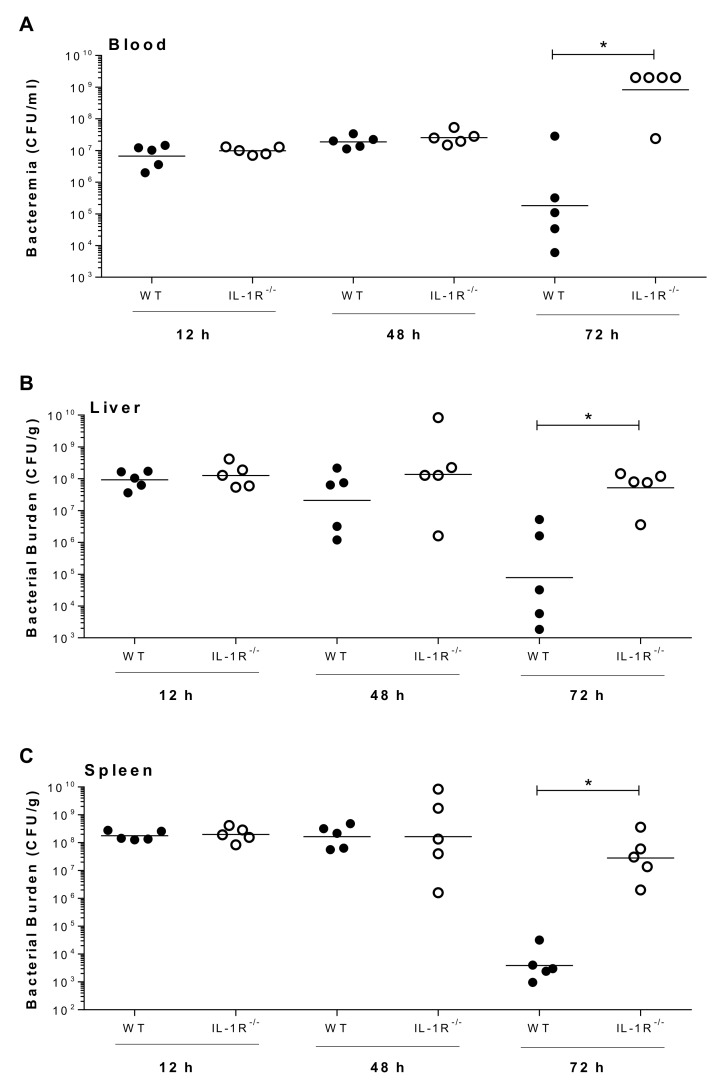
IL-1 is required for control of bacterial burden in blood, liver and spleen following SLY-negative *S. suis* strain 89-1591 infection. Bacterial burden in blood (**A**), liver (**B**), and spleen (**C**) of wild-type (WT) and IL-1R^-/-^ mice infected with strain 89-1591 (1 × 10^7^ CFU) 12 h, 48 h and 72 h post-infection. A blood bacterial burden of 2 × 10^9^ CFU/mL, corresponding to the average burden upon euthanasia, was attributed to euthanized mice. Data represent the geometric mean (*n* = 5). * (*p* < 0.05) indicates a significant difference between WT and IL-1R^-/-^ mice.

Since inflammation is required for initiation of bacterial clearance, bacterial burden was evaluated in the plasma, liver and spleen of wild-type and IL-1R^-/-^ mice infected with strain 89-1591. While no differences were observed in bacterial burden of wild-type and IL-1R^-/-^ mice 12 h and 48 h p.i., regardless of the organ, bacterial burden was significantly higher in the plasma, liver and spleen of IL-1R^-/-^ mice 72 h p.i. (*p* < 0.05) (Figure 10). Taken together, these data demonstrate that IL-1 signaling induced by a SLY-negative *S. suis* strain also contributes to pro-inflammatory mediator production and bacterial burden modulation involved in host survival.

## 3. Discussion

As an important porcine pathogen and zoonotic agent, *S. suis* possesses various components and virulence factors responsible for host cell activation and induction of an exacerbated inflammation [11,12,49]. This inflammatory response is composed of various mediators, including IL-1β, whose production was described to be promoted by elevated levels of secreted *S. suis* SLY [13,34]. However, ST25 strains, which are virulent and SLY-negative, account for nearly 50% of serotype 2 strains isolated from diseased pigs in Canada [5,10], yet their capacity to produce IL-1β and the mechanisms involved have been little studied.

Though somewhat less virulent in experimental infections than the highly virulent ST1 strains and the epidemic ST7 strain responsible for the 2005 human outbreak in China, strain 89-1591 causes significant disease and induced elevated IL-1β levels in liver and spleen that were of the same magnitude as those induced by the ST1 and ST7 strains [5,13]. Moreover, induced levels of IL-1β in plasma were similarly low between strains [13], which appears to be a characteristic of *S. suis* infection. Consequently, activation of splenic and liver immune cells might be responsible for its local production. In accordance, bmDCs, a commonly used model of conventional DCs [13,14,18,19,37], are an important source of *S. suis* ST1 and ST7-induced IL-1β (which both produce SLY under the conditions tested) [13]. This is also the case for the virulent SLY-negative ST25 strain, indicating that SLY alone is not the major component responsible for *S. suis*-induced IL-1β production. 

Strain 89-1591-induced IL-1β production from bmDCs involves a dual mechanism, depending on surface and intracellular recognition, similar to that described for *S. suis-*induced IFN-β [19]. By contrast to IFN-β production [19], however, recognition of surface bacterial LPs by TLR2 is important for IL-1β production. This immunostimulatory property of *S. suis* LPs and its recognition by TLR2 were previously described for other cytokines and chemokines [50,51]. Moreover, the lack of immunostimulatory properties of the *S. suis* LTA, including a lack of recognition by TLR2, confirms results published for other pro-inflammatory mediators [51]. Induction of IL-1β production by strain 89-1591 was also MyD88-dependent, TRIF-independent and TLR4-independent. However, the MyD88-dependancy of IL-1β and partial implication of TLR2, but lack of implication of TLR4, suggest the implication of other MyD88-dependent TLRs, of which there are at least half a dozen others [52]. Interestingly, MyD88 and TLR2, following recognition of LPs, are also required for induction of IL-1β by ST1 and ST7 strains, suggesting an evolutionary mechanism developed by the host to sense this pathogen and initiate an inflammatory response [13].

Alongside surface recognition by TLR2, strain 89-1591-induced IL-1β production was partially internalization-dependent, with residual levels of IL-1β being similar to those induced by lipopolysaccharide (which does not activate inflammasome assembly) when blocking internalization [53,54]. A similar internalization-dependent mechanism of IL-1β production was previously described for *Streptococcus pneumoniae* [55]. This internalization of *S. suis* has for consequence the release of nucleic acids within the cell, which might then be recognized by intracellular receptors, including endosomal TLRs. Indeed, it was previously demonstrated that the *S. suis* nucleic acids are recognized by the MyD88-dependent TLR7 and TLR9 in the case of IFN-β production [19]. In accordance, RNA and DNA from strain 89-1591 induce IL-1β, but only when complexed with a transfection reagent, herein possibly suggesting a role of TLR7 and TLR9. On the other hand, a TRIF-independent mechanism was observed, indicating that TLR3 would not be involved. Interestingly, though both nucleic acids induced IL-1β production, the stimulatory properties of DNA were significantly greater, suggesting a more important role of DNA-sensing receptors, which include the AIM2 inflammasome [52]. Indeed, DNA isolated from strain 89-1591 was the bacterial component majorly responsible for implication of AIM2 in induced IL-1β production, with a similar mechanism previously reported for *S. pneumoniae* [55]. *S. suis* nucleic acids are probably associated with various proteins when liberated in vivo. Given the immunostimulatory properties of most *S. suis* described proteins [11], this association might enhance the cell activating properties of nucleic acids, though future studies will be required to confirm the effect of this association on pro-inflammatory mediator production. 

Activation of TLR signaling leads to recruitment of the transcription factors NF-κB and MAPKs [16,17]. Results demonstrated that strain 89-1591-induced IL-1β production is dependent on the NF-κB, ERK, and JNK pathways, but is p38-independent. While the NF-κB and ERK pathways are also involved in IL-1β induced by SLY-positive ST1 and ST7 strains [13], this is the first study to describe a role of the JNK pathway. 

Together, the difference in IL-1β production induced by strains 89-1591 and P1/7 from bmDCs can be explained by the greater activation and participation of the NLRP3 and AIM2 inflammasomes for the former. Indeed, while NLRP3 inflammasome activation by SLY is almost solely responsible for *S. suis*-induced IL-1β production by SLY-positive *S. suis* [13,34], IL-1β production by a SLY-negative strain is dependent, at least partially, on phagocytosis susceptibility, resulting in intracellular localization of DNA activating the AIM2 inflammasome. Consequently, there exist at least two distinct mechanisms responsible for IL-1 production induced by *S. suis,* depending on capacity to produce SLY and on phagocytosis susceptibility.

Upon production, IL-1β binds the IL-1R, leading to cell activation, stimulation and secretion of diverse pro-inflammatory cytokines (positive feedback loop), among other effects [46]. In the case of SLY-positive *S. suis* ST1 infections, IL-1 signaling beneficially modulates the host innate immune response via increased production of other pro-inflammatory mediators required for control of bacterial burden in blood and organs, which, if unrestricted, causes host death [13]. Similar results were obtained in this study with the SLY-negative ST25 strain 89-1591, indicating that the role of IL-1 during *S. suis* infection is conserved and does not depend on SLY production. Importantly though, the role of IL-1, like that of type I IFN and possibly other mediators, appears to depend on the host inflammatory threshold not being exceeded. Indeed, inflammation induced by the highly virulent ST7 strain was reported to significantly exceed this threshold, which is critical for determining host outcome and survival, resulting in the role of IL-1 being difficult to discriminate [13]. Moreover, the role of 89-1591-induced IL-1 signaling on pro-inflammatory mediator production was only observed at later times points (48 h and 72 h p.i.). This is a consequence of IL-1 first having to be produced and secreted prior to binding its receptor and activating downstream signaling [46]. Moreover, the lower virulence of strain 89-1591, in comparison to the epidemic ST7 strain and highly virulent ST1 strains, influences the rapidity of the host response, impacting the observable effects. Taken together, these results clearly demonstrate that the inflammatory response induced by *S. suis* must be precisely balanced and controlled to be beneficial for host outcome and survival [13,17,19].

## 4. Material and Methods

### 4.1. Ethics Statement

This study was carried out in accordance with the recommendations of the guidelines and policies of the Canadian Council on Animal Care and the principles set forth in the Guide for the Care and Use of Laboratory Animals. The protocols and procedures were approved by the Animal Welfare Committee of the University of Montreal (protocol number rech-1570).

### 4.2. S. suis Strains and Growth Conditions

The strains used in this study are listed in Table 1. The *S. suis* serotype 2 ST25 strain 89-1591, isolated from a diseased pig with sepsis in Canada [56], was used throughout this study. This strain is highly encapsulated, SLY-negative and virulent [9,57]. In selected experiments, the highly virulent European *S. suis* serotype 2 ST1 reference strain P1/7 was used for comparison purposes. Isogenic mutants derived from strain 89-1591 were also included. *S. suis* was grown in Todd Hewitt broth (THB; Becton Dickinson, Mississauga, ON, Canada) as previously described [58], diluted in culture medium before experiments with cells and the final concentration (colony-forming units (CFU)/mL) determined by plating on THB agar (THA). For experimental mouse infections, bacteria were resuspended in THB.

### 4.3. Lipoteichoic Acid Preparation

Extraction and purification of LTA from strains 89-1591 and 89-1591Δ*lgt* was previously described [51,60].

### 4.4. Mice

MyD88^-/-^ (B6.129P2(SJL)-MyD88*^tm1.Defr^*/J), TRIF^-/-^ (C57BL/6J-Ticam1*^Lps2^*/J), TLR2^-/-^ (B6.129-Tlr2*^tmKir^*/J), TLR4^-/-^ (B6.B10ScN-Tlr4*^lps-del^*/JthJ), caspase-1^-/-^ (B6N.129S2-*Casp1^tm1Flv^*/J), NLRP3^-/-^ (B6.129S6-*Nlrp3^tm1Bhk^*/J), NLRP1^-/-^ (B6.129S6-*Nlrp1b^tm1Bhk^*/J), AIM2^-/-^ (B6.129P2-*Aim2^Gt(CSG445)Byg^*/J), NLRC4^-/-^ [61], and IL-1R^-/-^ (B6.129S7-*Il1r1^tm1Imx^*/J) mice on C57BL/6 background were housed under specific pathogen-free conditions alongside their wild-type counterparts. Mice were purchased from Jackson Research Laboratories (Bar Harbor, ME, USA), with the exception of NLRC4^-/-^ mice, which were originally generated by G. Núñez (University of Michigan, USA) [62].

### 4.5. Generation of Bone Marrow-Derived Dendritic Cells

The femur and tibia of wild-type and knock-out mice were used to generate bmDCs as previously described [14,63] in complete culture medium (RPMI-1640 supplemented with 5% heat-inactivated fetal bovine serum, 10 mM HEPES, 2 mM l-glutamine, and 50 µM 2-mercaptoethanol (Gibco, Burlington, ON, Canada) and complemented with 10% granulocyte-macrophages colony-stimulating factor). Cell purity was determined to be at least 85% CD11c^+^ [14,19,63]. Albeit this culture system cannot completely rule out the presence of other innate cells such as macrophages, it represents an enriched source of bmDCs.

### 4.6. S. suis Infection of Bone Marrow-Derived Dendritic Cells

All experiments were performed in the absence of endotoxin (lipopolysaccharide) contamination and under nontoxic conditions, the latter being evaluated by the lactate dehydrogenase release with the CytoTox 96® Non-Radioactive Cytotoxicity Assay (Promega, Madison, WI, USA). Cells were resuspended at 1 × 10^6^ cells/mL in complete medium and stimulated with the strains listed in Table 1 (1 × 10^6^ CFU/mL; initial multiplicity of infection = 1). Conditions used were based on those previously published [14,18]. For signaling pathway studies, cells were pretreated for 45 min with 10 μM NF-κB inhibitor JSH-23, 10 μM p38 inhibitor SB0203580, 25 μM MEK1/2 inhibitor U0126 or 10 μM JNK inhibitor SP600125 (all from Calbiochem/EMD Millipore, San Diego, CA, USA), or 5 μM cytochalasin D (Santa Cruz Biotech, Dallas, TX, USA), all in DMSO (Sigma-Aldrich), which served as the vehicle. At indicated times, supernatants were collected for cytokine measurements. Meanwhile, activation of cells with LTA was performed using 30 μg/mL and supernatants were collected 24 h later. For mRNA expression, cells were harvested in TRIzol (Invitrogen) 6 h following infection. Mock-infected cells served as negative controls. 

### 4.7. S. suis DNA and RNA Preparation and Transfection of Cells 

For bacterial DNA and RNA isolation, bacteria were grown to mid-log phase. Total RNA was extracted using the Aurum Total RNA Mini Kit (Bio-Rad) according to the manufacturer’s instructions, including treatment with DNase I. For DNA preparation, bacteria were harvested in 10 mM Tris, 1 mM EDTA, pH 8.0, and treated with 10% SDS and 20 mg/mL proteinase K (Sigma-Aldrich) for 1 h at 37 °C. DNA was isolated using phenol/chloroform/isoamyl alcohol (Sigma-Aldrich) [64]. After isolation, bacterial DNA was treated with 10 mg/mL RNase A (Roche) for 30 min at 37 °C. Cells were transfected with 1 μg of RNA or DNA complexed with DOTAP liposomal transfection reagent (Sigma-Aldrich) as previously described [19,64,65].

### 4.8. Preparation of Heat-Killed S. suis and Bacterial Lysates

Heat-killed *S. suis* were prepared as described in [66]. Briefly, a bacterium was grown to mid-log phase and incubated at 60 °C for 45 min. Cultures were subcultured on blood agar plates at 37 °C for 48 h to confirm absence of bacterial viability. Heat-killed *S. suis* were resuspended in cell culture medium at a concentration of 2 × 10^9^ CFU/mL prior to bmDC stimulation. Bacterial lysates were prepared as described [64] and cells transfected with 5 μg of lysates complexed with DOTAP liposomal transfection reagent.

### 4.9. IL-1β Quantification in Cell Culture Supernatants

Levels of IL-1β in cell culture supernatants were measured by sandwich enzyme-linked immunosorbent assay (ELISA) using pair-matched antibodies from R&D Systems (Minneapolis, MN, USA) according to the manufacturer’s recommendations.

### 4.10. Determination of Cell mRNA Expression by RT-qPCR

Cell mRNA was extracted according to the manufacturer’s instructions (TRIzol). RNA purity was assessed by spectrophotometric quantification and integrity verified by electrophoresis on denaturating agarose gel. cDNA was generated using the Quantitect cDNA Synthesis Kit (Qiagen, Mississauga, ON, Canada) with 500 ng of RNA pretreated with DNase. Real-time polymerase chain reaction (qPCR) was performed on the CFX-96 Touch Rapid Thermal Cycler System (Bio-Rad), using 250 nM of primers (Integrated DNA technologies), the SsoFast Evagreen Supermix Kit (Bio-Rad) and 20 ng of cDNA. No template controls were included and all samples were run in triplicate. The cycling conditions were 3 min of polymerase activation at 98 °C, followed by 40 cycles at 98 °C for 2 s and 57 °C for 5 s. Melting curves were generated after each run to confirm the presence of a single PCR product. The sequences of primers used in this study are shown in Table 2 and were verified to have reaction efficiencies between 90% and 110%. The reference genes *Atp5b* and *Gapdh*, determined to be the most stably expressed using the algorithm geNorm, were used to normalize data. Fold changes in gene expression were calculated using the quantification cycle threshold (Cq) method using the CFX software manager v.3.0 (Bio-Rad). Samples from mock-infected cells served as calibrators.

### 4.11. S. suis Mouse Model of Infection

Six-week-old male and female wild-type C57BL/6 and IL-1R^-/-^ mice were used. Animals were acclimatized to standard laboratory conditions with unlimited access to water and rodent chow [36]. These studies were carried out in strict accordance with the recommendations of and approved by the University of Montreal Animal Welfare Committee guidelines and policies, including euthanasia to minimize animal suffering, applied throughout this study when animals were seriously affected, since mortality was not an end point measurement. Strain 89-1591 was administered at a dose of 1 × 10^7^ CFU by intraperitoneal inoculation to groups of 15 mice. Survival was evaluated and mice were monitored twice daily until 72 h post-infection (p.i.) and twice thereafter until 10 days p.i. 

### 4.12. Measurement of Plasma, Liver and Spleen Pro-Inflammatory Mediators

For kinetics of IL-1β production, wild-type mice were inoculated with strain 89-1591 or the vehicle (sterile THB) as described above. At 12 h, 48 h and 72 h p.i., blood was collected by intracardiac puncture following euthanasia and anti-coagulated with EDTA (Sigma-Aldrich) as previously described [57]. Plasma supernatants were collected following centrifugation at 10,000 × *g* for 10 min, 4 °C. For liver and spleen, extraction buffer was prepared using complete Mini, EDTA-free, protease inhibitor cocktail tablets (Roche Diagnostics GmbH, Mannheim, Germany) according to the manufacturer’s instructions, and organs homogenized using a POLYTRON PT 1200E system bundle (Kinematica, Lucerne, Switzerland). Homogenate supernatants were collected following centrifugation at 10,000 × *g* for 10 min, 4 °C, and stored at −80 °C. Levels of IL-1β were determined by ELISA as described, while IL-6, IFN-γ, CCL3, and CXCL9 were measured using a custom-made cytokine Bio-Plex Pro™ assay (Bio-Rad) according to the manufacturer’s instructions. Acquisition was performed on the MAGPIX platform (Luminex®) and data analyzed using the Bio-Plex Manager 6.1 software (Bio-Rad).

### 4.13. Measurement of Blood, Spleen, and Liver Bacterial Burden

Wild-type and IL-1R^-/-^ mice were infected with strain 89-1591 as described above and blood bacterial burden was assessed 12 h, 48 h, and 72 h p.i. by collecting 5 μL of blood from the caudal tail vein. For liver and spleen, organs were collected and homogenized as described above. Bacterial burden was determined by plating appropriate dilutions on THA. 

### 4.14. Statistical Analyses 

Normality of data was verified using the Shapiro–Wilk test. Accordingly, parametric (unpaired *t*-test) or non-parametric tests (Mann–Whitney rank sum test), where appropriate, were performed to evaluate statistical differences between groups. Log-rank (Mantel–Cox) tests were used to compare survival between wild-type and IL-1R^-/-^ mice. Each test was repeated in at least three independent experiments. *p* < 0.05 was considered as statistically significant.

## 5. Conclusions

**Figure 11:**
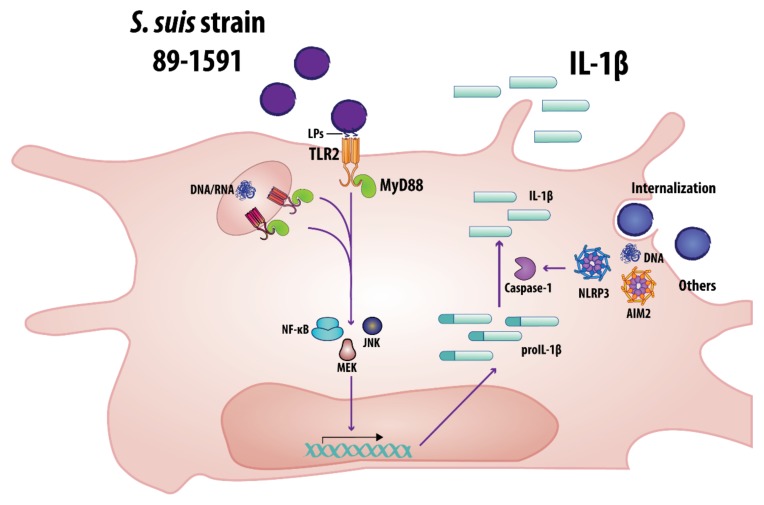
Proposed model of the mechanisms involved in SLY-negative virulent *S. suis* strain 89-1591-induced IL-1β production by bmDCs. Bacterial recognition by bmDCs requires MyD88-dependent signaling and partial involvement of TLR2 via recognition of lipoproteins (LPs). Following internalization, bacterial DNA and RNA can also induce IL-1β production. *S. suis* recognition then leads to activation of the NF-κB, MEK and JNK pathways. Finally, activation of the NLRP3 and AIM2 inflammasomes, the latter by bacterial DNA following elevated internalization, is involved in caspase-1-dependent processing of IL-1β.

This study demonstrates that an elevated production of IL-1β in internal organs is a characteristic of *S. suis* infection caused by virulent strains, regardless of SLY production. More precisely, IL-1β production by SLY-negative *S. suis* is a consequence of conserved bacterial components being recognized, namely LPs via TLR2, and following internalization, nucleic acids, possibly via TLR7 and TLR9. Interestingly, and different from what was previously reported, IL-1β processing does not solely depend on SLY: we demonstrate a novel SLY-independent mechanism whereby intracellularly located *S. suis* DNA is recognized by the AIM2 inflammasome. Based on these results, a model of the mechanisms involved in SLY-negative *S. suis* strain 89-1591-induced IL-1β production by bmDCs is proposed (Figure 11). Finally, *S. suis*-induced IL-1 plays a beneficial role during the systemic infection by initiating the inflammatory cascade involved in bacterial control and clearance, and this regardless of SLY production. Consequently, this study will help to better understand the underlying mechanisms involved in *S. suis*-induced inflammation and disease.

## Figures and Tables

**Table 1:** *S. suis* serotype 2 strains used in this study.

Strain	General Characteristics	Reference
89-1591	Virulent North American ST25 strain isolated from a case of pig sepsis in Canada	[56]
89-1591Δ*cpsF*	Non-encapsulated isogenic mutant derived from 89-1591; in frame deletion of *cpsF* gene	[40]
89-1591Δ*lgt*	Isogenic mutant strain derived from 89-1591; in frame deletion of *lgt* gene	[51]
P1/7	Classical highly virulent ST1 strain isolated from a pig with meningitis in the United Kingdom, used for comparison	[59]

**Table 2:** Primers used in this study.

Primer Name	Sequence (5′ – 3′)
*Atp5b*	F: ACC AGC CCA CCC TAG CCA CC R: TGC AGG GGC AGG GTC AGT CA
*Gapdh*	F: CCC GTA GAC AAA ATG GTG AAG R: GAC TGT GCC GTT GAA TTT G
*Il1b*	F: AGG TCA AAG GTT TGG AAG CA R: TGA AGC TAT GGC AAC TG

## Abbreviations

AIM2	absent in melanoma 2
bmDC	bone marrow-derived dendritic cell
CCL	C-C motif chemokine ligand
CFU	colony-forming unit
CXCL	C-X-C motif chemokine ligand
DC	dendritic cell
DMSO	dimethylsulfoxide
IFN	interferon
IL	interleukin
IL-1R	interleukin-1 receptor
JNK	c-Jun N-terminal kinase
LP	lipoprotein
LTA	lipoteichoic acid
MAPK	mitogen-activated protein kinase
MEK1/2	mitogen-activated protein kinase 1/2
MyD88	myeloid differentiation primary response 88
NF-κB	nuclear factor kappa B
NLR	NOD-like receptor
NLRC	NLR family CARD domain-containing protein
NLRP	NLR family pyrin domain-containing
NOD	nucleotide oligomerization domain
PCR	polymerase chain reaction
p.i.	post-infection
PRR	pattern recognition receptor
p38	p38 mitogen-activated protein kinase
SLY	suilysin
ST	sequence type
THA	Todd Hewitt broth agar
THB	Todd Hewitt broth
TLR	Toll-like receptor
TRIF	TIR-domain-containing adapter-inducing interferon-β
